# Influence of cerebral blood vessel movements on the position of perivascular synapses

**DOI:** 10.1371/journal.pone.0172368

**Published:** 2017-02-15

**Authors:** Miguel Urrecha, Ignacio Romero, Javier DeFelipe, Angel Merchán-Pérez

**Affiliations:** 1 Department of Mechanical Engineering, Universidad Politécnica de Madrid, Madrid, Spain; 2 IMDEA Materials Institute, Getafe, Madrid, Spain; 3 Laboratorio Cajal de Circuitos Corticales, Centro de Tecnología Biomédica, Universidad Politécnica de Madrid, Pozuelo de Alarcón, Madrid, Spain; 4 Instituto Cajal, Consejo Superior de Investigaciones Científicas, Madrid, Spain; 5 Departamento de Arquitectura y Tecnología de Sistemas Informáticos, Universidad Politécnica de Madrid. Pozuelo de Alarcón, Madrid, Spain; Universidad de Zaragoza, SPAIN

## Abstract

Synaptic activity is regulated and limited by blood flow, which is controlled by blood vessel dilation and contraction. Traditionally, the study of neurovascular coupling has mainly focused on energy consumption and oxygen delivery. However, the mechanical changes that blood vessel movements induce in the surrounding tissue have not been considered. We have modeled the mechanical changes that movements of blood vessels cause in neighboring synapses. Our simulations indicate that synaptic densities increase or decrease during vascular dilation and contraction, respectively, near the blood vessel walls. This phenomenon may alter the concentration of neurotransmitters and vasoactive substances in the immediate vicinity of the vessel wall and thus may have an influence on local blood flow.

## Introduction

**Fig 1:**
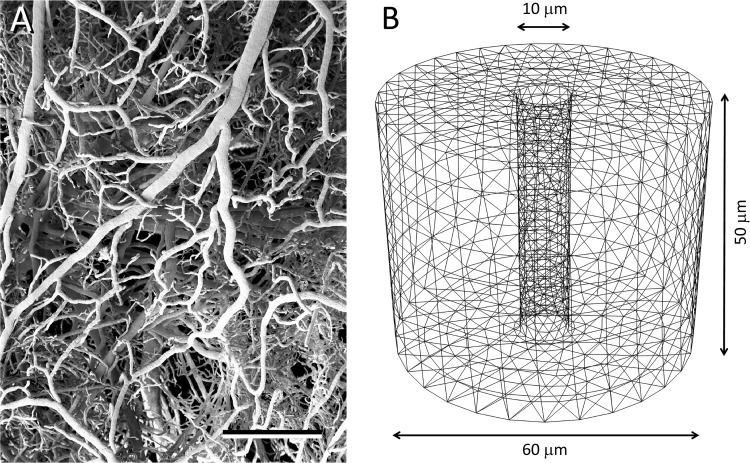
Blood vessel architecture and finite element model. (A) Scanning electron micrograph of a vascular corrosion cast illustrating the density and complexity of cortical vasculature in the rat. Cortical surface is to the top of the figure. Scale bar: 500 μm. From Merchan-Perez and DeFelipe, unpublished material. See also S1 Movie. (B) The finite element model represents a cylinder of gray matter traversed from top to bottom by a small blood vessel. The blood vessel is considered to be a hollow cylinder with a diameter of 10 μm. The whole model diameter is 60 μm, and its height is 50 μm. Gray matter is assumed to be a linearly-elastic isotropic material. The body has been meshed into approximately 15,000 constant strain tetrahedral elements.

**Fig 2:**
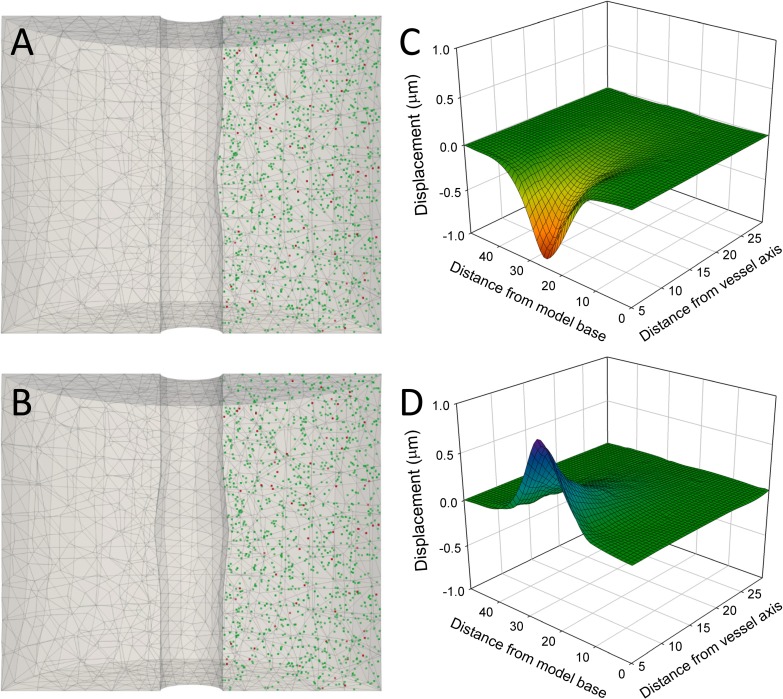
Three-dimensional model of a brain blood vessel and the tissue and synapses that surround it. The model in (A) and (B) represents a cylinder of brain tissue crossed by a blood vessel from top to bottom. Excitatory (glutamatergic) and inhibitory (GABAergic) synapses have been represented as green and red points, respectively. The cylinder height is 50 μm and its diameter is 60 μm. The diameter of the blood vessel at rest is 10 μm. In (A), the maximum contraction of the central part of the blood vessel has been represented (the diameter decreases to 8 μm). In (B), maximum dilation of the central part of the blood vessel has been represented (the diameter increases to 12 μm). No displacement was allowed at the external boundary of the model. The displacements of the synapses that were randomly distributed within the model are represented in (C) and (D) during maximal contraction, and dilation, respectively. See also S2 and S3 Movies.

Coupling between local blood perfusion and neuronal activity is central to normal brain function. Neurovascular coupling is a complex phenomenon by which changes in neuronal activity are followed by vessel constriction or dilation that cause variations in local cerebral blood flow and metabolism [1]. These changes in blood supply and tissue metabolism also provide the basis for functional neuroimaging methods [2–4]. Cortical blood vessels form a dense and highly interconnected network [5,6] (Fig 1A), and it has been shown that there are differences between cortical areas and layers [7–9]. Although it has recently been shown that the mechanical properties of brain tissue limit vascular dilation [10], little attention has been paid to the mechanical changes that blood vessel movements may induce in the microstructure of perivascular tissue. In fact, gray matter is a highly deformable tissue, with a Young’s modulus of about 3 kilopascals (kPa), so it is much softer than, for example, muscle (~7 kPa), skin (~85 kPa), or liver and kidney (~190 kPa) [11]. Here we have used a simulation approach to study the effects of blood vessel constriction and dilation on the surrounding tissue, especially on the displacement of neighboring synapses. We have used a finite element mechanical model [12] consisting of a cylinder of grey matter that is crossed from top to bottom by a small blood vessel with a diameter of 10 μm (Fig 1B). Points representing the position of synapses have been randomly distributed around the blood vessel with a density of 1 synapse/μm^3^ based on 3D electron microscope data obtained by combined focused ion beam milling and scanning electron microscopy [13,14] (S1 Movie). Blood vessel movements have been simulated based on data regarding contractions and dilations that take place in living blood vessels, and assume the caliber of the vessel to be 8 μm during maximal contraction and 12 μm during maximal dilation [15–17] (Fig 2).

## Materials and methods

### Geometry of the model

The simulations presented here have been developed with a finite element mechanical model [12] of a small blood vessel surrounded by gray matter. The blood vessel is represented as a hollow cylinder with a radius of *r* = 5 μm. The boundaries of the gray matter volume are defined by the following elements: (i) the cylindrical wall of the capillary vessel, (ii) an external cylindrical wall coaxial with the blood vessel and with radius *R* = 30 μm, and (iii) two parallel planes perpendicular to the vessel axis, separated by 50 μm. Gray matter is assumed to be a linearly-elastic isotropic material. The synapses that are present within the gray matter are considered as 0-dimensional points without material properties, with each of them representing the centroid of an actual synapse. The centroids were randomly located inside the volume, with a density of 1.00 synapse/μm^3^ [13,14].

### Finite element model

The body has been meshed into approximately 15,000 constant strain tetrahedral (CST) elements. The analyses have been run with a general purpose in-house finite element code developed in the Computational Mechanics Group of the School of Mechanical Engineering at the Universidad Politécnica de Madrid. A standard finite element formulation has been employed, and therefore the displacement field over the volume was piecewise linear. The analyses run were also based on small strain assumptions and employed the linearized elasticity equations. In order to simulate the contractions and dilations that take place in living blood vessels [15–17], a displacement field was set on the capillary wall, imposing a maximum 20% decrease of the radius during contraction and a maximum 20% increase during dilation over the transversal mid-sectional plane of the cylinder. In this way, the radius of the blood vessel in the model mid-plane ranged between 4 and 6 μm from maximal contraction to maximal dilation, respectively. The displacement field decays linearly in both directions of the cylinder axis from the mid-sectional plane, vanishing at an axial distance of more than 10 μm. No displacements were allowed at the boundaries of the model.

### Postprocessing

Since the centroids of the synapses are purely geometrical objects, the displacement of these particular entities must be interpolated from the displacement field obtained as the solution of the model. The randomness of the distribution of centroids implied the need for a point-location algorithm, such that each synapse motion could be interpolated from the displacement of the four vertices of the tetrahedron where each centroid was originally embedded. This algorithm, based on Quad-Octree theory, was implemented from the free-access C++ ANN library [18].

### Analytical displacement equation on the transversal mid-plane

Additionally, the analytical expression of the displacement field on the transversal mid-plane of the model (where displacements were maximal) can be formulated [19]. This was done by taking into account the axisymmetric boundary conditions, the isotropy of the material, and the assumption of having a plane strain state on the mid-plane. The displacement solution can be expressed as:

$$u(r)=C_{1}+C_{2}\frac{1}{r}$$

This equation is adjusted for every quasistatic step, knowing:

$$\left\{ \begin{array}{l} u(r)=u(t)\\ \;u(R)=0 \end{array}\right.\;r=5,\;R=30,\;u(t)\in [-1,+1]$$

## Results

**Fig 3:**
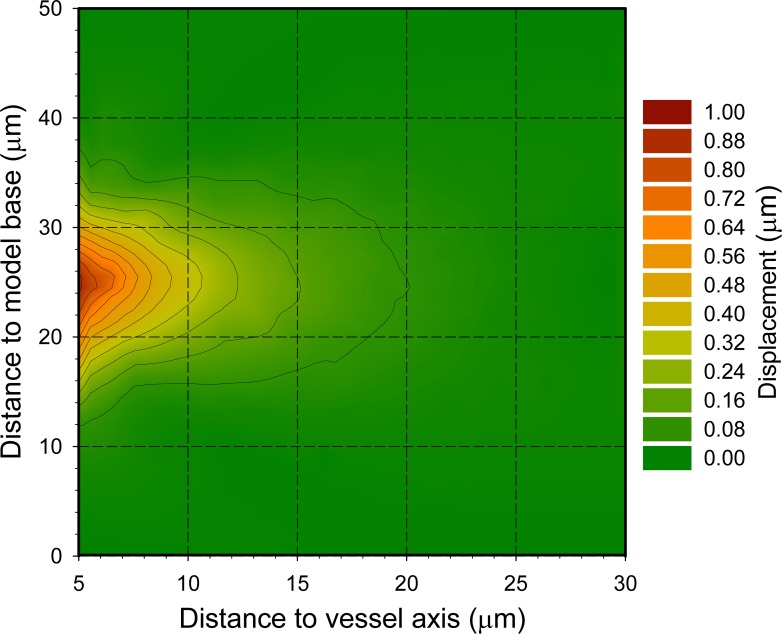
Blood vessel movements and displacement of synapses. The graph represents a displacement field at maximum contraction of the blood vessel. Contraction takes place at the central part of the blood vessel, as in Fig 2A. The displacements of randomly-located synapses around the blood vessel have been color-coded as in Fig 2C. Note that maximum displacements take place close to the vessel wall at the model mid-plane and they rapidly decrease both axially and radially, so that displacements are close to zero in most of the model volume.

**Fig 4:**
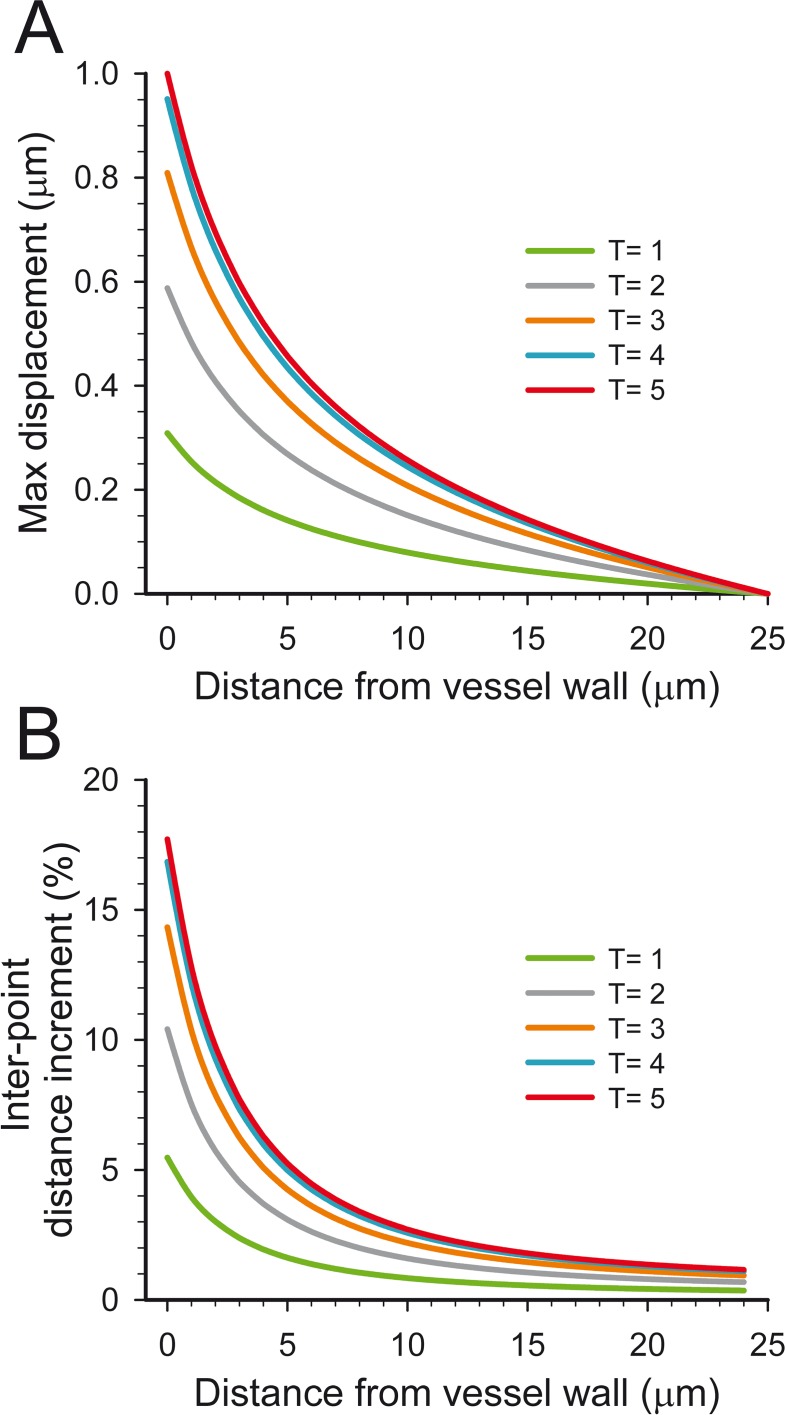
Maximum displacement of points during blood vessel contraction or dilation. To compute these curves, 26 points were used. The first point was located on the maximum deformation point of the vessel wall (at the model mid-plane) and the rest were radially distributed at regular intervals (1 μm) until the external boundary of the model was reached. The maximum displacement of the blood vessel wall was 1 μm from the resting state during contraction (inwards) or dilation (outwards). No displacement in the external boundaries of the model was allowed. (A) Maximum absolute displacement of points at regular time intervals (T = 1 to T = 5) until maximum contraction or dilation of the blood vessel is reached (T = 5). (B) Inter-point distance increment as a percentage of the resting-state distance (1 μm). Points were arranged in the same way as in (A), and the distances between each point and its outward neighbor were recorded at the same time intervals. Since the behavior of the model is symmetric, these increments represent an increase of inter-point distances during blood vessel contraction and a decrease during dilation.

Synapses located at different positions in the model were affected differently by constriction or dilation of the middle region of the blood vessel. Maximum displacements were found close to the vessel wall at the model mid-plane and they rapidly decreased axially and radially (Figs 2 and 3, S2 and S3 Movies). In fact, only 9.82% of the points were displaced more than 0.1 μm in the whole model when the maximal radial displacement of the vessel wall was 1 μm. That is, tissue displacements were confined to the close proximity of the region of the blood vessel that actually moves. The behavior of the model was symmetric, so the displacements during vessel contraction (Fig 2A and 2C) were inverse to the displacements during dilation (Fig 2B and 2D). To further characterize the perivascular displacements caused by blood vessel movements, we also analytically calculated the displacements of regularly distributed points at the model mid-plane (where changes were maximal)[19]. Results also showed a rapid decrease of displacement from the vessel wall to the outer boundary of the model (Fig 4). For example, an absolute maximum displacement of 1 μm at the blood vessel wall was approximately halved every 5 μm as we moved perpendicularly away from the model axis in the model mid-plane (Fig 4A). If relative displacements are considered, the maximum inter-point distance increment was about 18% for points that were close to the vessel wall, decreasing to about 5% at points that were 5 μm away from the vessel wall (Fig 4B).

## Discussion

It can be concluded that the spatial distribution of most synapses and the cellular processes in the neuropil that are relatively far from the blood vessels will not be mechanically affected by blood vessel movements, while synapses and cellular processes that are close to the vessel wall will be subject to displacements that halve approximately every 5 microns as we move away from the vessel wall (Fig 4). This latter effect may have several consequences. First, it may help to spread or restrict the diffusion of perivascularly released signals. Increased synaptic activity releases multiple signals that eventually cause a vasoactive response either directly or mediated by astrocytes [20–22]. Glutamate is one of the main signals triggering neurovascular responses [23–25]. This neurotransmitter can evoke the release of nitric oxide from neurons [23,26] or it can initiate Ca^++^ rises in glial perivascular endfeet that eventually cause the release of vasoactive substances [25,27–29]. Second, perivascular endfeet surround the vessel wall, so they are located in the region where synaptic displacements are maximal. Thus, they could be sensitive to the increase or decrease of synaptic density in the surrounding neuropil during vascular dilation and contraction, respectively. For example, the compressed tissue around a dilated vascular region in the cortex will show a transient increase in synaptic density that will result in a higher concentration of released glutamate, even though no increase in synaptic activity takes place at all. Released glutamate will help maintain (or reverse) vascular dilation through its activity on local vascular endfeet. It must be noted that the time scale of synaptic events is in the order of milliseconds, while vascular events take place on a time scale of seconds or longer, so changes in the relative density of synapses located near blood vessels will be maintained long enough to increase (or decrease) the concentrations of vasoactive substances in the immediate vicinity of the vessel wall.

Our model is based on the local changes that take place in living blood vessels under different stimuli, as previously described and imaged [15–17]. Although these local contractions and dilations have been described in vessels with diameters between 5 and 30 microns, we have limited our analysis, for the sake of simplicity, to an idealized blood vessel segment with a diameter of 10 microns at rest. This would correspond to a parenchymal arteriole (see, for example,[15,16,25,30]). Our model, however, is scalable to larger (or smaller) vessel calibers. In addition, distortions affecting long vascular segments can be modeled by applying the analytical displacement equation on the transversal mid-plane (see Methods) to the whole vessel segment under deformation. Finally, our approach can also be applied to any kind of vessel deformation, either active or passive, so it may be used in other scenarios, provided the magnitude of vessel wall displacements and the density of synapses are known.

## Supporting information

S1 Movie A small blood vessel and the surrounding brain tissue reconstructed from serial electron microscopic images obtained by FIB/SEM. A total of 358 serial images, separated by 20 nm, have been stacked and aligned so the blood vessel (orange) and synaptic junctions (excitatory in green; inhibitory in red) can be segmented and reconstructed in 3D. The volume of the imaged tissue is 12.57 x 10.66 x 7.16 cubic microns.
(AVI)

S2 Movie Three-dimensional model of a brain blood vessel and the tissue and synapses that surround it. The model represents a cylinder of brain tissue crossed by a blood vessel from top to bottom. Asymmetric and symmetric synapses have been represented as green and red points, respectively. The cylinder height is 50 μm and its diameter is 60 μm. The diameter of the blood vessel at rest is 10 μm. The video sequence shows the maximum contraction of the central part of the blood vessel, when the diameter decreases to 8 μm, and the maximum dilation, when the diameter increases to 12 μm.
(AVI)

S3 Movie Displacements of synaptic junctions surrounding a blood vessel. The model represents the displacements of synapses that are randomly located around a blood vessel during blood vessel movements. The diameter of the vessel during maximal contraction is 8 μm and 12 μm at maximal dilation.
(AVI)
